# Diversity of soil seed bank and influencing factors in the nascent wetland of the Yellow River Delta

**DOI:** 10.3389/fpls.2023.1249139

**Published:** 2023-09-01

**Authors:** Tao Zhu, Qing Fang, Luhao Jia, Yuhan Zou, Xuehong Wang, Chenyu Qu, Junbao Yu, Jisong Yang

**Affiliations:** The Institute for Advanced Study of Coastal Ecology, Key Laboratory of Ecological Restoration and Conservation of Coastal Wetlands in Universities of Shandong, Ludong University, Yantai, Shandong, China

**Keywords:** the Yellow River Delta, soil seed bank, nascent wetland, species diversity, effecting factors

## Abstract

Soil seed bank is the growth and reproduction source of vegetation community, playing an important role in vegetation establishment, succession and renewal, biodiversity maintenance. This study has selected the nascent wetland in the Yellow River Delta (YRD) formed in 1996 as study area and investigated the diversity and key influencing factors of soil seed bank diversity. The study results show that: (1) The soil seed bank in the study area has a simple structure, containing relatively few species. A total of five plant species, which belong to four families and five genera, were found in this bank, with *Phragmites australis* and *Suaeda salsa* being the dominant plants. (2) All species are herbs without woody species. One herb is annual herb and the others are perennial herbs. (3) From the sea to the river, the changes rules of the overall density and diversity of the seed bank are not obvious. (4) The dispersal distance from salt and freshwater has a significant influence on the density of the soil seed bank but has no significant influence on the diversity. Meanwhile, the soil salt content has a significant negative influence on the diversity of seed banks. (5) Aboveground vegetation did not closely relationship with diversity of soil seed bank. All above results can provide basic data and scientific evidence for the conservation of vegetation communities in the nascent wetlands and vegetation restoration in the degraded wetlands in the YRD.

## 1 Introduction

The seed bank is a key component of ecosystem resilience, playing an important role in the maintenance of species diversity worldwide (Kalamees and Zobel, 2002; Bradbury et al., 2016; Vandvik et al., 2016; Poschlod and Rosbakh, 2018). Moreover, the seed bank can recruit species lost from aboveground vegetation (Schmiede et al., 2009; Kalamees et al., 2011), and it is used as a potential resource to protect and restore species diversity (Thompson and Fenner, 2000; Schmiede et al., 2009; Ma et al., 2018). Therefore, it can promote a healthy regional ecosystem and improve biodiversity in the region (Beas et al., 2013; Wang et al., 2016; Wang et al., 2019), playing an important role in vegetation succession and renewal, biodiversity maintenance, and wetland restoration. (Liu, 2005; Guan et al., 2019). Correctly understanding the composition structure, distribution pattern, and key influencing factors of regional soil seed banks will become a decisive factor in the construction and restoration of vegetation communities.

The soil seed bank is influenced by various factors, the including environmental factors and anthropogenic factors (Baldwin et al., 2010; Ruano et al., 2015; Benvenuti and Mazzoncini, 2021). Environmental factors mainly include water regime, soil properties and aboveground vegetation and so on (Bai et al., 2014). The environmental factors have a relatively complex influence on the wetland plant seed bank (Gonçalves et al., 2020) and can affect the size and distribution of the seed bank and the germination characteristics of various species (Crosslé and Brock, 2002; Metzner et al., 2017). For example, the soil pH value is a key factor influencing the diversity of seed banks and plant communities (Ma et al., 2017b; Ren et al., 2023), increases in soil pH may lead to a decrease in seed bank activity (Ma et al., 2017a; Li et al., 2019). The soil salinity and alkalinity can affect the composition and scale of the effective seed bank and restrict the renewal of the wetland vegetation community (Zhao et al., 2022). Underground water level (Kaiser and Pirhofer-Walzl, 2015; Feng et al., 2021) can affect the content of water-soluble salt, thus influencing the diversity of soil seed banks and vegetation coverage degree. The process of sediment deposition and soil erosion conditions can alter the illumination and temperature required by seed germination (Hilary et al., 2016; Wang et al., 2016), thus affecting the soil seed bank. In the process, the burial depth of sediment plays a crucial role (Limón and Peco, 2016; Egawa, 2017). Drought and flood frequency can interfere with the stable living environment of the seed bank, thus suppressing the abundance and multiplicity of the wetland seed bank, with terrestrial species having higher abundance in drought environments and aquatic and amphibian species having superior abundance in flooded environments (Bao et al., 2018; Schneider et al., 2020).

The soil banks could be also affected by human activities, such as grazing and other disturbance (Chu et al., 2019). The diversity of wetland seed banks in a disturbed area is normally greater than that diversity in a not-disturbed area (Middleton, 2016). Also, the diversity decreases with the increase in the distance from the disturbed area. That is, the diversity changes monotonously with the variation of the disturbance gradient (Peterson and Baldwin, 2004). In the nascent wetland of the YRD, soil seed bank was mainly affected by environmental factors because human activities in this area are negligible.

The Yellow River Delta wetland is the youngest wetland in China. This wetland has a rich biodiversity and plays an important role in the habitat protection of wetland animals, especially birds (Cui et al., 2009). However, under the dual effects of tidal currents and runoff, the nascent wetland ecosystem in the Yellow River Delta is relatively fragile, with a frequent succession of plant communities and a drastic fluctuation of biodiversity (Guan et al., 2019). Thoroughly understanding the nascent wetland soil seed bank in the Yellow River Delta is of great significance for correctly interpreting the establishment, development, and succession of plant communities in the region, improving biodiversity and promoting a healthy and stable nascent wetland. Therefore, this paper has taken the soil seed bank in the new-born wetland of the Yellow River Delta after the diversion of the Yellow River in 1996 as the research object, aim to 1) explore the species and diversity of the soil seed bank in the nascent wetland, 2) find the key influencing factors of the soil seed bank in the nascent wetland of the Yellow River Delta. Results would provide data support for the establishment and succession of plant community in the nascent wetland of the Yellow River Delta, and provide scientific evidence for the protection and management of the new-born wetland.

## 2 Materials and methods

### 2.1 Study site

The Yellow River Delta wetland (118°33′-119°207′E, 37°35 ′-38°12′N) is the best preserved, youngest, and widest wetland ecosystem in the warm temperature zone of China (Chen et al., 2016; Yu et al., 2016). With a temperate continental monsoon climate, this area has an average annual temperature of 11.7-12.6°C and average annual precipitation of 540-600mm, which primarily concentrates in Summer. Also, loam is the dominant soil in this area (Wang et al., 2015). Its coastal area has a high level of salinization, with few plant species and vegetation varieties.

### 2.2 Sample collection

**Figure 1:**
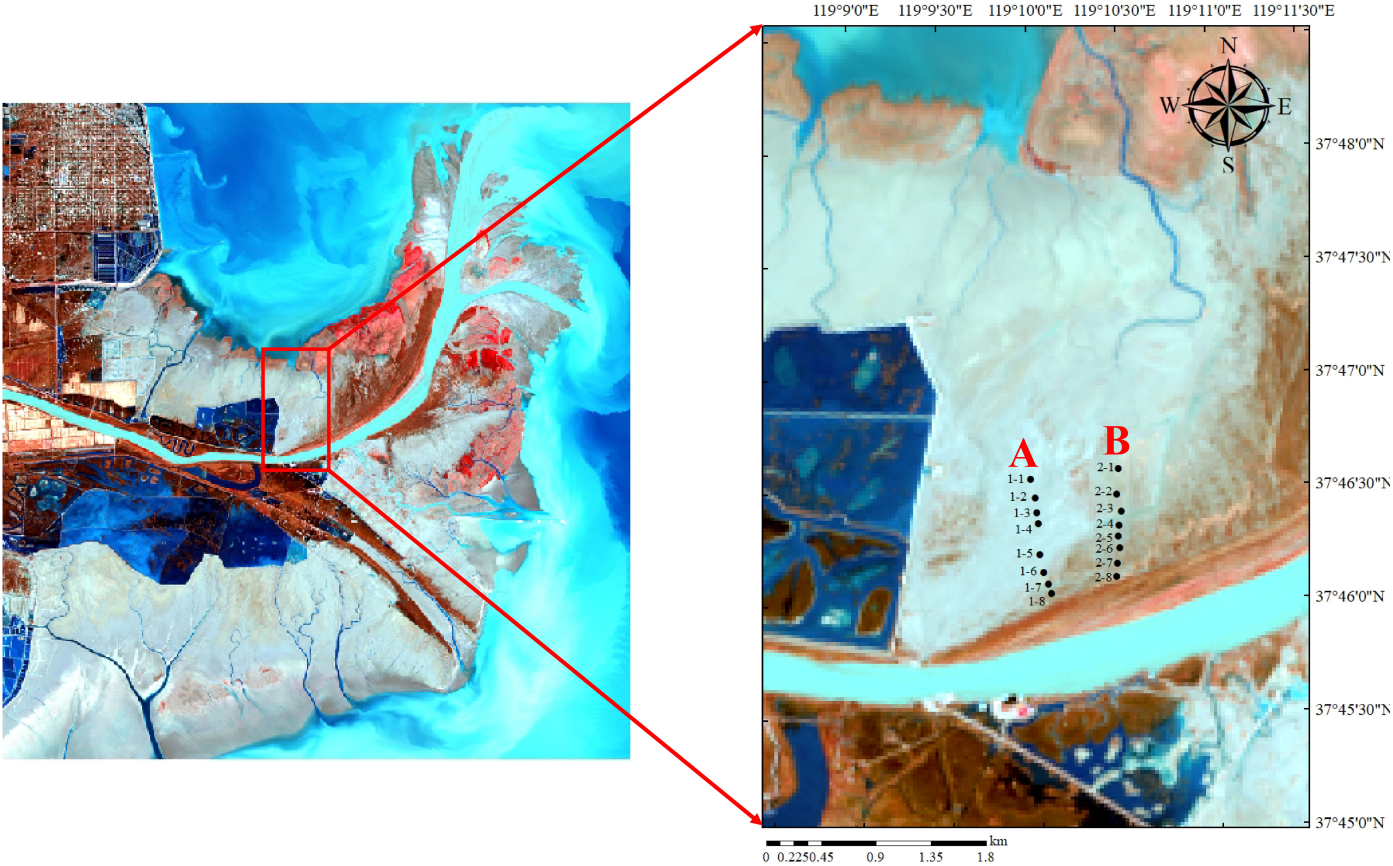
Study area of nascent wetland in the Yellow River Delta. **(A, B)** are belt transects from west to east.

In January 2022, samples of soil seed banks were collected in the nascent wetland of the Yellow River Delta. Along the silting direction of the nascent wetland of the Yellow River Delta, two parallel belt transects (**Figure 1** belt transect A and B) with representative vegetation communities and lower frequency of anthropogenic disturbances perpendicular to the direction of the Yellow River entering the sea were set up on the north bank of the Yellow River, and samples were collected in a way that combines belt transect and sample point method. There was a space range of 500-700m between the two adjacent belt transects. Within each belt transect, there was an interval of 50-150m between two adjacent sample points. With a soil collection tool, a soil sample of 10 cm×10cm was collected at each point with a sampling depth of 20cm. Soil samples were collected at four layers with depths of 0-5cm, 5-10cm, 10-15cm, and 15-20cm, respectively. Three repetition sampling points were set at each sample point, with a distance of 5-10m between two adjacent repetition sampling points. Then, soil samples collected from the same layer of the repetition sampling points were mixed into a sample.

### 2.3 Germination of seed bank

A method of soil sample concentration was applied in this experiment (Heerdt et al., 1996). First, the collected seed bank samples were dried in sunlight. And then the large plant fragments and stones were removed using a 4 mm sieve, and then the soil was filtered with a 2 mm sieve (Ma et al., 2017b). After the residual plant tissues and stones in the samples were carefully removed, the seed bank samples were placed in a pot (30cm×20cm×10cm) for germination in a greenhouse in LuDong University. First, a 5cm layer of vermiculites were placed at the bottom of the experimental soil to hold water (Li, 2022). Second, prepared seed bank samples were evenly spread on the vermiculites layer. During the germination process, all pots were watered regularly to ensure adequate moisture. When seedlings appeared in the pots, they were identified. The species name and number of seedlings per pot were recorded. The identified seedlings were removed, meanwhile, unknown seedlings were transplanted to separately pots to grow until it could be identified (Gao et al., 2021). After all seedlings were identified, the soil samples were turned over to continue germination until no seedlings appeared for 2 weeks (Ma et al., 2011).

### 2.4 Measurement of soil properties

Three soil cores (d = 3.6 cm, h = 10 cm) were randomly taken next to seed samples quadrats and mixed into a sample to determine soil properties. Soil electrical conductivity and pH value were measured with a conductivity meter with glass electrodes and a pH meter, respectively. Soil total nitrogen content was measured with the Kjeldahl method (Stanley et al., 2019), soil total organic matter content was measured with the potassium dichromate external heating method (Kopáček et al., 2001), and soil total phosphorus content was measured with the ultraviolet spectrophotometric method (Kopáček et al., 2001; Miller and Arai, 2016; Mikajlo et al., 2023).

### 2.5 Data analysis

In this paper, the Shannon-Wiener diversity index (SWI) (Zou et al., 2021), Patrick richness index (PRI) (Courkamp et al., 2022), Jaccard similarity index (JCI) (Zhang et al., 2017), and Total seed density (TSD) were used to reflect the diversity of seed banks.

Patrick richness index:


R=S

Shannon-Wiener diversity index:


$$H^{\prime }=-\sum \text{Pi }lnPi$$

Jaccard similarity index:


$$J(A,B)=\frac{\left|A\cap B\right|}{\left|A\cup B\right|}$$

Where, *S* represents the number of plant species; *p_i_* refers to the ratio of the number of individual plant species to the total number of the plant community; *A* represents the composite set of ground vegetation species; and *B* is the composite set of the soil seed bank.

Microsoft Excel 2023, Origin 9.2, and GraphPad 9.3 software were used to perform the data sorting, statistical analysis, and chart drawing. One-way ANOVA by Duncan’s multiple range test (P< 0.05) was used to assess the differences in seed composition, distribution and diversity along transect A and B. In addition, a correlation analysis method was applied to analyze the relationship between the soil seed bank and its influencing factors, with all applied data being averages of three values repeatedly measured.

## 3 Results

### 3.1 Composition and density of the soil seed bank

#### 3.1.1 Species composition of the soil seed bank

**Figure 2:**
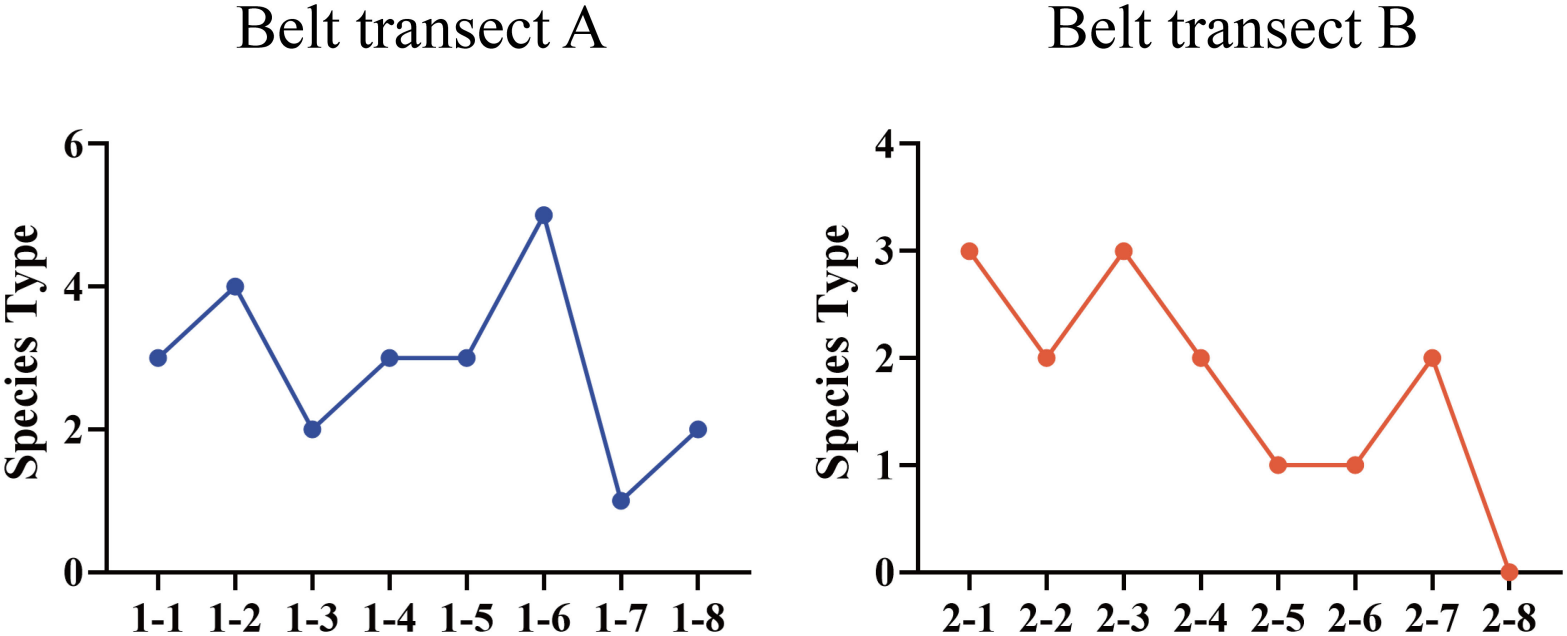
Species richness of each sample point of belt transects A and B.

**Figure 3:**
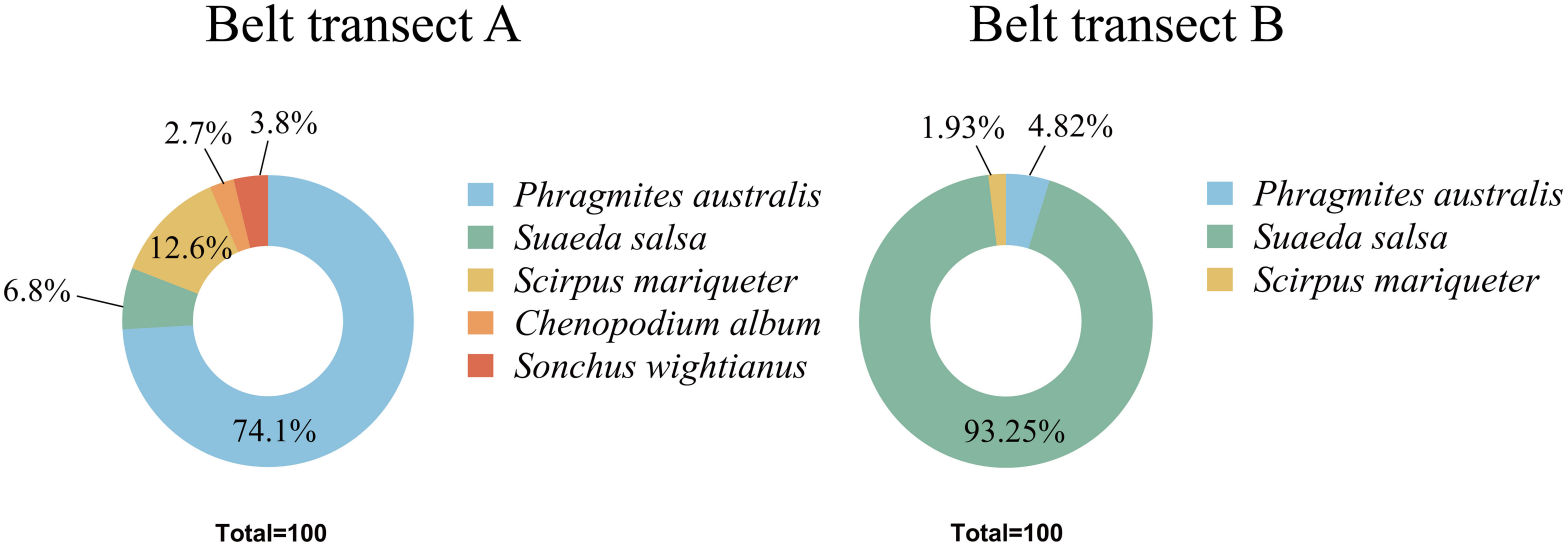
Species proportions of belt transects A and B.

Five higher herb plant species, belonging to four families and five genera, were germinated in the soil seed bank in the nascent wetland of the Yellow River Delta. From the coast to the river, the plant species in the seed bank present a trend of fluctuating variation (**Figure 2**). There is a significant difference (*p ≤* 0.05) between the soil seed bank species in the belt transects of A and B. The species richness of belt transect A is higher than that of the belt transect B. **Figure 3** shows that all the five plant species mentioned above are found in the belt transect A. Among these species, *Phragmites australis*, followed by *Scirpus mariqueter, Suaeda salsa*, and *Sonchus brachyotus*, accounts for a highest proportion of 74.1%, and Chenopodium album accounts for a lowest proportion of only 2.7%. Species of *Phragmites australis*, *S. salsa* and *S. mariqueter* were found in the belt transect B. Among these species, *S. salsa* accounts for an absolutely dominant proportion (93.25%), and *S. mariqueter* accounts for a lowest proportion (1.93%).

Species varieties in the soil seed bank vary with the soil depth (**Table 1**). Species varieties in belt transect A shows a trend of increasing first and decreasing then. There are three plant species found within the soil layer range of 0-5cm, and five plant species found within the soil layer range of 5-10cm, as well as the soil layer range of 10-15cm. Meanwhile, there are four plant species found within the soil layer range of 15-20cm. With the increase of soil depth, the species number in the belt transect B presents a trend of fluctuating variation. There is only one species of *S. salsa* within the soil layer range of 15-20cm, with no presence of *Chenopodium album* and *S. brachyotus* within the whole soil depth.

**Table 1:** Plant species in belt transect A and B.

	0-5		5-10		10-15		15-20	
	A	B	A	B	A	B	A	B
*Phragmites australis*	+	+	+	–	+	+	+	–
*Suaeda salsa*	+	+	+	+	+	+	+	+
*Scirpus mariqueter*	+	+	+	+	+	+	+	–
*Chenopodium album*	–	–	+	–	+	–	+	–
*Sonchus brachyotus*	–	–	+	–	+	–	–	–

+ means the species is present; - means the species is not present.

#### 3.1.2 Density of soil seed bank

**Figure 4:**
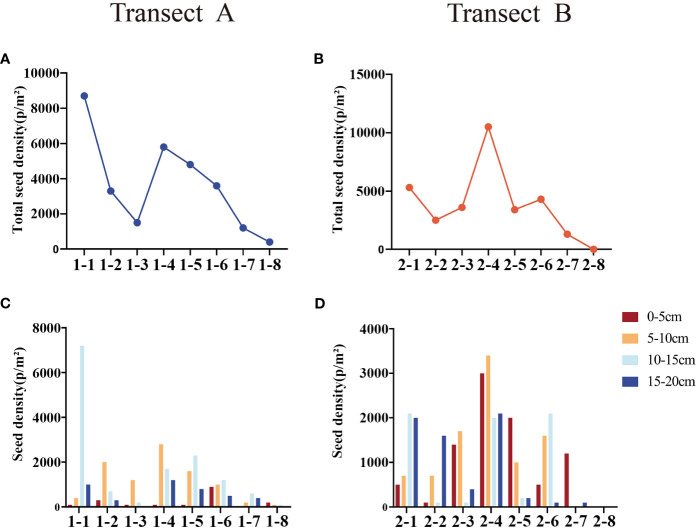
Germination densities of seed banks at different depths in each belt transect. **(A, B)** are total seed density in transect A and B, respectively; **(C, D)** are seed density of each sample in transect A and B, respectively.

Generally, the seed densities in both belts transects are similar. The overall seed bank density decreased from the sea to the river (**Figure 4**). From the sea to the river, the seed density of the belt transect A presents a nonlinear downward trend, while the seed density of the belt transect B presents a pattern of increasing first and decreasing then. The belt transect A has a largest seed bank density in the coastal mudflat zone, and the belt transect B has a largest seed bank density in the area of saline and freshwater interaction. The overall seed bank density presents a trend of decrease. There are significant differences among the densities of soil seed banks at different depths. The densities of belt transect A’s seed bank at different depths vary significantly, and the densities of belt transect B’s seed bank within the depth ranges of 0-10cm and 10-20cm are significantly different from each other (**Table 2**). The soil seed density in the belt transect A within the depth range of 0-5cm is significantly lower than that in the belt transect B (*p<0.05*), and the seed density of the belt transect A at the depth of 10-15cm significantly higher than that density of the belt transect B (*p<0.05*). while the soil seed densities in the both belts transects at the depth range of 5-10cm and 15-20cm are similar (*p>0.05*).

**Table 2:** Seed density of different buried depth in belt transect A and B (Unit: kp/m²).

	0-5cm	5-10cm	10-15cm	15-20cm
Belt transect A	0.225 ± 0.101^d^	1.163 ± 0.334^b^	1.75 ± 0.822^a^	0.525 ± 0.157^c^
Belt transect B	1.088 ± 0.365^a^	1.138 ± 0.393^a^	0.825 ± 0.364^b^	0.813 ± 0.325^b^

kp/m² means thousand grains/m², the lower-case letters indicate significant and non-significant differences at p< 0.05.

### 3.2 Diversity of the soil seed bank in the nascent wetland

**Figure 5:**
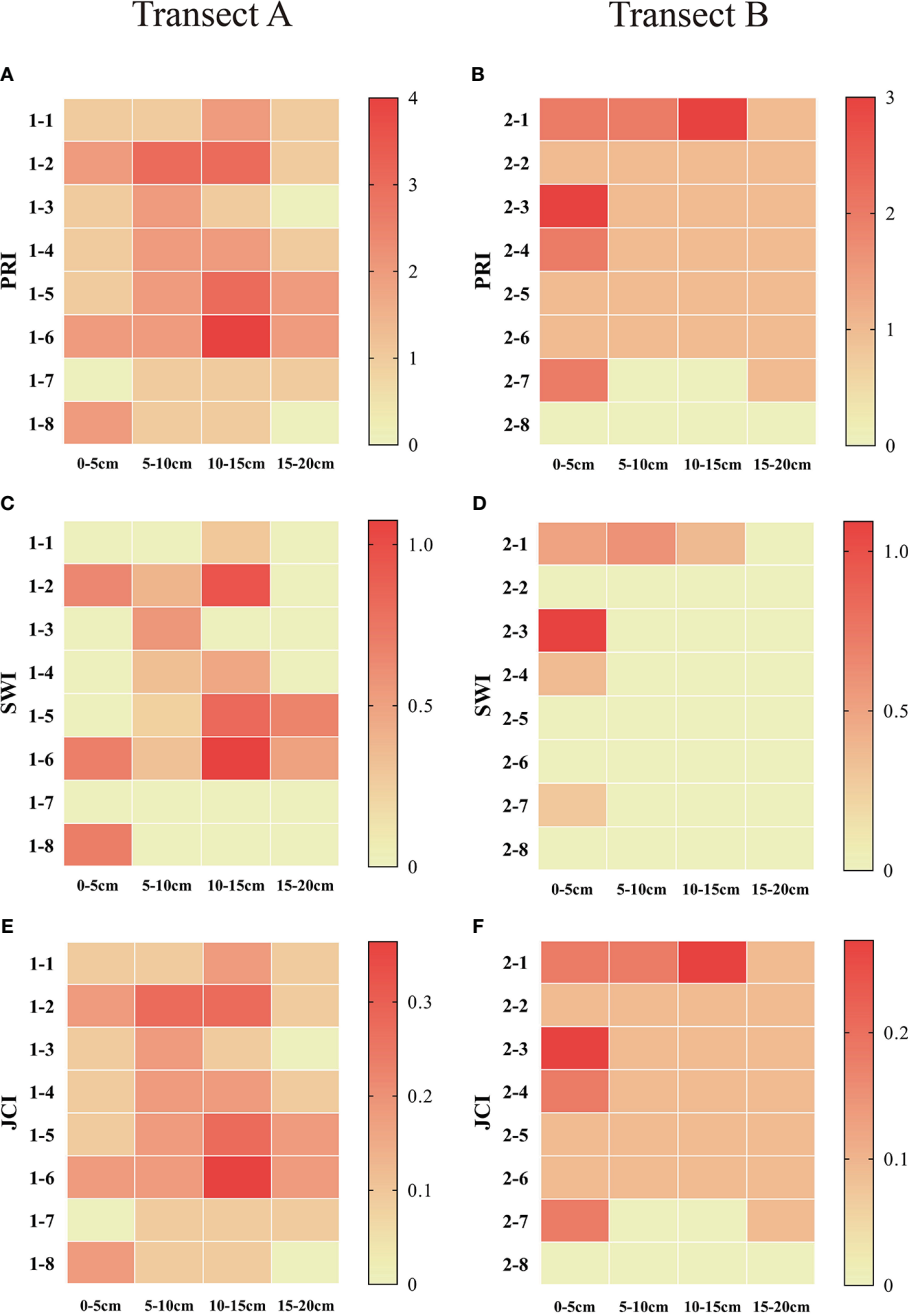
Heat map of diversity index of seed bank at different depths in each belt transect. **(A–F)** represent Patrick richness index (PRI), Shannon-Wiener diversity index (SWI) and Jaccard similarity index in belts transects A and B, respectively.

The results of diversity indices show that, from the sea to the river, the PRI, SWI, and JCI of the soil seed bank present similar trends of variation, all showing a trend of increasing first and decreasing then (**Figure 5**). The diversity index of belt transect A has a higher value than that of belt transect B. In addition, the diversity of soil seed bank at different depth are also different. The SWI, PRI, and JCI indices of belt transect A at a depth of 5-15cm have a highest value, while the indices of belt transect B have a highest value at a depth of 0-5cm. Also, these three indices of belt transect A and B have a lowest value at a depth of 15-20cm.

### 3.3 Influencing factors of soil seed bank diversity in nascent wetland

#### 3.3.1 Soil properties in each belt transect

**Figure 6:**
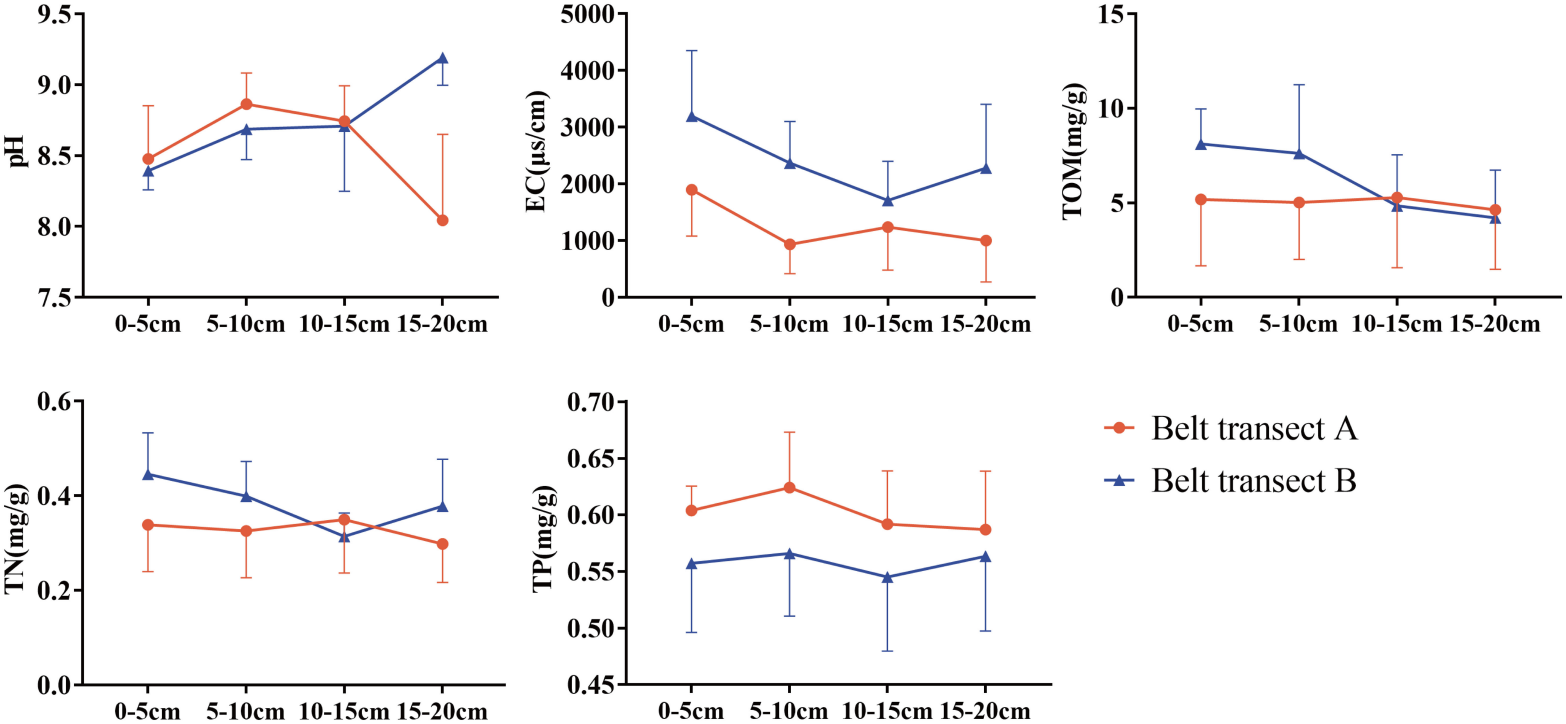
Soil physicochemical properties at different depths of each belt transect.

The results of soil properties show that, from the sea to the river, pH gradually decreases as a whole, EC and TP show an overall gradual increase trend, while TOM and TN present a fluctuating variation. Different belt transect has different soil properties. Generally, the EC of belt transect B has a higher value than the EC of belt transect A. Meanwhile, the TP content of belt transect A is higher than the TP content of belt transect B. Content of the TOM in both belts transects gradually becoming equal with the increase of depth (**Figure 6**). Soil properties also vary with the soil depth. Within the belt transect A, pH, and TP present a trend of increasing first and gradually decreasing then with the increase of soil depth, and EC peaks at the soil depth of 0-5cm, and then gradually decreases and then tends to stabilize. Meanwhile, TN increases first and then decreases with the increase of soil depth, peaking at the soil depth of 10-15cm. However, with the increase of soil depth, TOM content does not change significantly. Within the belt transect B, pH increases with the soil depth, and EC and TN decrease first and then increase with the increase of soil depth, reaching their minimum values at the soil depth of 10-15cm. TOM content decreases with the increase of soil depth, while TP presents a trend of increasing first, decreasing then, and increasing again with the increase of soil depth, with no significant differences among contents at different soil depths.

#### 3.3.2 Key factors influencing density and diversity of soil seed bank

**Figure 7:**
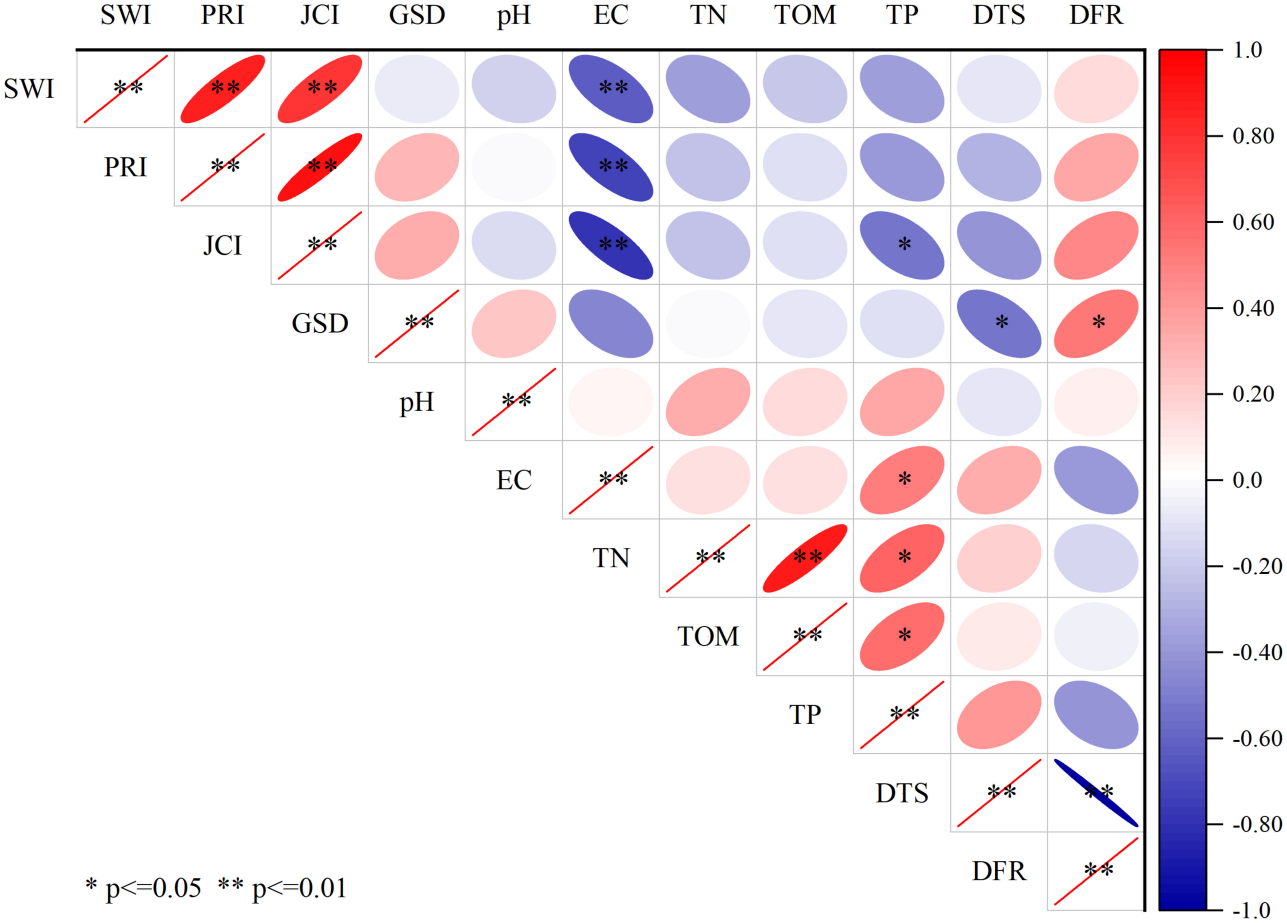
Correlation analysis diagram of species indices of the soil seed bank and environmental factors.

Results indicate that environmental factors have a significant influence on the compositional and diversity of seed banks (**Figure 7**). The seed bank density is significantly correlated with the distance of dissemination, with a significant negative correlation between seed bank density and DTS and a significant positive correlation between seed bank density and DFR. The plant diversity indices have no significant relationships with DFR and DTS, however, they have significant relationships with EC which is directly affected by the distance to the sea and the river. the density and diversity do not show obvious relationship with soil nutrients. Above results suggest that distance to the sea and the river rather than soil nutrients determine the density and diversity of soil seed bank in the YRD.

## 4 Discussion

### 4.1 Species and diversity of the soil seed bank

In order to ensure the succession of species, plants often propagate through seeds to reduce their survival risks, thus maintaining the long-term existence of species (Childs et al., 2010; Metzner et al., 2017). Annual and perennial plants usually adopt different reproductive strategies. Annual plants mainly regenerate by seeds, while perennial plants can reproduce multiple times depending on their underground roots (Ida et al., 2013). Therefore, the species composition and density of seed banks are closely related to the species composition of surface aboveground vegetation (Capers, 2003; Liu, 2005; Chu et al., 2019). In the study of urban soil seed bank, herb plant species accounted for most of the soil seed banks, herb species were about one third of herb species in the aboveground vegetation communities, while woody species only were tenth of the woody plant species in the aboveground vegetation communities (Zhao et al., 2023). In alpine wetland, species and diversity of soil seed bank were significantly correlated with aboveground vegetation species, but seed bank dynamics do not change with aboveground vegetation during wetland degradation (Zhao et al., 2021). In order to ensure the continuation of species, plants often propagate through seeds to reduce their survival risks, thus maintaining the long-term existence of species (Childs et al., 2010; Childs et al., 2010; Metzner et al., 2017). In our study, 5 plant species of the soil seed bank were all herbs without woody plant, including one annual herb and four perennial herbs. *S. salsa* is annual herb which only regenerate by seeds. *T P. australis*, *S. mariqueter*, *C. album* and *S. brachyotus* are perennial herbs most depending on asexual reproduction around their aboveground vegetation communities. There are 11 species in aboveground vegetation, including 9 herb species and 2 woody species (**Table 3**). Species and diversity of soil seed bank did not have close relationship with aboveground vegetation (**Figures 4E, F**). Tiny seeds of herb species nearby the river could be not spread over the woody plant to establish a new population by wind or water (Wang et al., 2015), which mainly resulted in species composition of soil seed bank of the nascent wetland in the YRD was significant different with the aboveground vegetation. The formation of a seed bank is influenced by various factors (Baldwin et al., 2010; Ruano et al., 2015; Benvenuti and Mazzoncini, 2021).

**Table 3:** Species in soil seed bank and aboveground vegetation.

Species of soil seed bank	Species of aboveground vegetation
*Phragmites australis*, *Suaeda salsa* *Chenopodium album*, *Sonchus brachyotus* *Scirpus mariqueter*	*Phragmites australis*, *Chenopodium album*, *Sonchus brachyotus, Suaeda salsa*, *Glycine soja* *Calamagrostis pseudophragmites*, *Tamarix chinensis* *Typha laxmannii*, *Salix matsudana Koidz* *Imperata cylindrica*, *Miscanthus sacchariflorus*

### 4.2 Factors influencing soil seed bank

Many factors could affect species and density of soil seed bank, such as surface configuration (Havrdová et al., 2015), climate (Andrea et al., 2015), vegetation characteristics (Ma et al., 2017a), soil conditions (Ma et al., 2017b; Seibert et al., 2018), water regime (Anneke et al., 2018), and so on. In areas with good conditions, soil seed banks have a complex species composition and a higher density. On the contrary, in areas with disturbed factors, soil seed banks have a relatively simple composition and lower density (Zhao et al., 2022). To a certain extent, the fluctuation of soil water and salt contents will interfere with the balance of density and species composition of the soil seed bank. Also, the soil nutrient content will influence the seed variety and density of the soil seed bank with different influencing degrees. Some studies have shown that the species richness and seed density of wetland seed banks can be slightly affected by soil nutrient enrichment (Miao and Zou, 2009). Meanwhile, it is argued that the density condition of soil seed banks has a close relationship with the soil nutrient contents of nitrogen, phosphorus, and potassium (He et al., 2016).

In the estuary wetland, the initial vehicle of dispersal is wind (Chen et al., 2019; Zhu et al., 2022). When seeds fall to the ground, they will dispersal again faraway under the action of tides and surface runoff (Kim et al., 2022; Wang et al., 2022). Consequently, the seed dispersal distance and the consequent changes in the relevant factors have greatly impacted density and diversity of the seed bank. Existing studies have shown that dispersal distance has a significant influence on density of soil seed bank, but they did not show significant impact on the species composition (Wei et al., 2017). In the Tianjin coastal wetland, soil seed bank size is huge, but it contains relatively few species, which are primarily saline-alkaline tolerant plant, such as *P. australis*, *S. salsa*, *and Aeluropus littoralis* (He et al., 2014). Results in this paper are consistent with above results. In our study, density of soil seed bank in the new-formed wetland is great, but the species is only 5 herbs.

Among these factors, environmental factors can affect the compositional structure and density of soil seed banks. In areas with good environmental factors, soil seed banks have a complex compositional structure and a high richness of species. On the contrary, in areas with bad environmental factors, soil seed banks have a simple compositional structure and low richness of species (Zhao et al., 2022). To a certain extent, the fluctuation of soil water and salt contents will interfere with the balance of density and species composition of the soil seed bank. Also, the soil nutrient content will influence the seed variety and density of the soil seed bank, with different influencing degrees. Some studies have shown that the species richness and seed density of wetland seed banks can be slightly affected by soil nutrient enrichment (Miao and Zou, 2009). Meanwhile, it is argued that the density condition of soil seed banks has a close relationship with the soil nutrient (He et al., 2016). The results of this research show that EC has a certain influence on TSD, while there is no significant relationship between soil nutrients and TSD. The results of this study are slightly different from the results reported in the previous literature. The primary reason is that the salt content can exert a main inhibiting effect on the growth and manifestation of the plant community, thus further affecting the formation of the seed bank (Li and Xu, 2009).

In this study, an interesting finding is distance to the sea or river determining the density of soil seed bank, and salinity greatly affecting the diversity of soil seed bank(p ≤ 0.01) (**Figure 7**). But there is no significant relationship between soil nutrients and density of seed bank. This are halfway similar to the results reported in the previous literature (Li and Xu, 2009; Erfanzadeh et al., 2010; Bai et al., 2014; Gao et al., 2018). The primary reason is that the salinity can exert a main inhibiting effect on the growth and manifestation of the plant community in the estuary wetlands, thus further affecting the formation of the seed bank. In this paper, diversity of soil seed bank with different buried depth are also affected by soil salt (**Figure 6**). Our results have fully verified the influence of salt water and freshwater interactions on the spatial heterogeneity of soil seed banks.

*Spartina alterniflora* is an invasive species with great negative effects in the YRD. However, in this study, no *S. alterniflora* seeds were found in the soil bank in the nascent wetland. This is probably due to the fact that samples were obtained from areas with few disturbances, and distance to *S. alterniflora* community is long enough, limiting the dispersal and establishment of invasive species.

## 5 Conclusions

Plant establishment and succession are important for a new and restored wetland and herb species appeared preferentially in the wetlands. In this study, species, diversity and influencing factors of soil seed banks in the nascent wetland of the Yellow River Delta were investigated, with the following conclusions:

(1) The soil seed bank of the nascent wetland in the Yellow River Delta has relatively few species and low diversity, and diversity of soil seed bank do not have greatly relationship with aboveground vegetation.
(2) Species diversity was higher in the area with interaction of salt water and fresh water.
(3) The seed densities are significant different at different buried depths.
(4) Seed dispersal distance has a significant impact on the density of the soil seed bank but has no significant influence on the diversity of the seed bank. soil salinity is the key factors determined diversity of soil seed bank.

## Data availability statement

The original contributions presented in the study are included in the article/supplementary material. Further inquiries can be directed to the corresponding author.

## Author contributions

TZ and XW: conceptualization, methodology, investigation, experiment, and writing – original draft preparation. XW, JSY, and JBY: supervision, investigation, and funding acquisition. QF, LJ, and CQ: investigation, experiment, and data curation. All authors contributed to the article and approved the submitted version.

## References

[B1] Andrea M. Simone P. Giulietta B. Graziano R. Thomas A. Robin J. P.. (2015). Climate warming could increase recruitment success in glacier foreland plants. Ann. Bot. 116, 907–916. doi: 10.1093/aob/mcv101 26133689 PMC4640126

[B2] Anneke D. R. Olivier R. Mathilde D. Hatsadong T. Bounsamay S. (2018). Weed seed dispersal via runoff water and eroded soil. Agric., Ecosyst. Environ. Appl. Soil Ecol. 256, 48–502. doi: 10.1016/j.agee.2018.05.026

[B3] Bai J. H. Huang L. B. Gao Z. Q. Lu Q. Q. Wang J. J. Zhao Q. Q.. (2014). Soil seed banks and their germination responses to cadmium and salinity stresses in coastal wetlands affected by reclamation and urbanization based on indoor and outdoor experiments. J. Hazard. Mater. 280, 295–303. doi: 10.1016/j.jhazmat.2014.07.070 25173982

[B4] Baldwin A. H. Kettenring K. M. Whigham D. F. (2010). Seed banks of *Phragmites* australis-dominated brackish wetlands: Relationships to seed viability, inundation, and land cover. Aquat Bot. 93, 163–169. doi: 10.1016/j.aquabot.2010.06.001

[B5] Bao F. Elsey-Quirk T. De Assis M. A. Pott A. (2018). Seed bank of seasonally flooded grassland: experimental simulation of flood and post-flood. Aquat. Ecol. 52, 93–105. doi: 10.1007/s10452_017_9647_y

[B6] Beas B. J. Smith L. M. LaGrange T. G. Stutheit R. (2013). Effects of sediment removal on vegetation communities in Rainwater Basin playa wetlands. J. Environ. Manage. 128, 371–379. doi: 10.1016/j.jenvman.2013.04.063 23786876

[B7] Benvenuti S. Mazzoncini M. (2021). “Active” Weed seed bank: soil texture and seed weight as key factors of burial-depth inhibition. Agronomy 11, 210. doi: 10.3390/agronomy11020210

[B8] Bradbury D. Tapper S. Coates D. McArthur S. Hankinson M. Byrne M. (2016). The role of fire and a long-lived soil seed bank in maintaining persistence, genetic diversity and connectivity in a fire-prone landscape. J. Biogeogr. 43, 70–84. doi: 10.1111/jbi.12601

[B9] Capers R. S. (2003). Macrophyte colonization in a freshwater tidal wetland (Lyme, CT, USA). Aquat. Bot. 77, 325–338. doi: 10.1016/j.aquabot.2003.08.001

[B10] Chen A. Sui X. Wang D. S. Liao W. G. Ge H. F. Tao J. (2016). Landscape and avifauna changes as an indicator of Yellow River Delta Wetland restoration. Ecol. Eng. 86, 162–173. doi: 10.1016/j.ecoleng.2015.11.017

[B11] Chen F. Q. Wu Y. Zhang M. Ma Y. R. Xie Z. Q. Chen C. (2019). Secondary seed dispersal in hydro-fluctuation belts and its influence on the soil seed bank. River Res. Appl. 35, 405–413. doi: 10.1002/rra.3411

[B12] Childs D. Z. Metcalf C. J. E. Rees M. (2010). Evolutionary bet-hedging in the real world: empirical evidence and challenges revealed by plants. P. Roy Soc. B-Biol Sci. 277, 3055–3064. doi: 10.1098/rspb.2010.0707 PMC2982066 20573624

[B13] Chu H. Zhang C. P. Dong Q. M. Shang Z.H. Degen A. A. Yang X.X. (2019). The effect of grazing intensity and season on the soil seed bank and its relation with above-ground vegetation on the alpine steppe. Agr Ecosyst. Environ. 285, 106622. doi: 10.1016/j.agee.2019.106622

[B14] Courkamp J. S. Meiman P. J. Paschke M. W. (2022). Indaziflam Reduces Seed Bank Richness and Density but not Sagebrush-Grassland Plant Diversity. Rangeland Ecol. Manage. 84, 31–44. doi: 10.1016/j.rama.2022.05.005

[B15] Crosslé K. Brock M. A. (2002). How do water regime and clipping influence wetland plant establishment from seed banks and subsequent reproduction? Aquat. Bot. 74, 43–56. doi: 10.1016/S0304-3770(02)00034-7

[B16] Cui B. S. Tang N. Zhao X. S. Bai J. H. (2009). A management-oriented valuation method to determine ecological water requirement for wetlands in the Yellow River Delta of China. J. Nat. Conserv. 17, 129–141. doi: 10.1016/j.jnc.2009.01.003

[B17] Egawa C. (2017). Wind dispersal of alien plant species into remnant natural vegetation from adjacent agricultural fields. Glob. Ecol. Conserv. 11, 33–41. doi: 10.1016/j.gecco.2017.04.008

[B18] Erfanzadeh R. Hendrickx F. Maelfait J. Hoffmann M. (2010). The effect of successional stage and salinity on the vertical distribution of seeds in salt marsh soils. Flora 205, 442–448. doi: 10.1016/j.flora.2009.12.010

[B19] Feng L. Liu J. T. Han G. X. Zhang Q. H. Peng L. (2021). Effects of groundwater level change on soil seed bank characteristics in coastal wetlands of the Yellow River Delta. Acta Ecol. Sin. 41, 3826–3835. doi: 10.5846/stxb202006281666

[B20] Gao G. F. Li X. Y. Chen Y. Shi S. M. Ge X. L. (2021). Germination of soil seed banks of two communities in Mata Lake Wetland. J. Qilu Univ. Technol. 35, 17–22. doi: 10.16442/j.cnki.qlgydxxb.2021.05.003

[B21] Gao R. R. Wei X. Y. He Z. Zhao R. H. Wang K. Yang X. J.. (2018). Soil salt and NaCl have different effects on seed germination of the halophyte Suaeda salsa. J. Plant Nutr. Soil Sc. 181, 488–497. doi: 10.1002/jpln.201700544

[B22] Gonçalves B. G. Ribeiro L. M. Dias D. S. Mazzottini-dos-Santos H. C. Martins C. D. P. S. Lopes P. S. N.. (2020). Embryo responses to extreme water events provide insights into the behavior of Butia capitata (Arecaceae) seed banks during hydration cycles. Environ. Exp. Bot. 169, 103904. doi: 10.1016/j.envexpbot.2019.103904

[B23] Guan B. Chen M. Elsey-Quirk T. Yang S. S. Shang W. T. Li Y. Z.. (2019). Soil seed bank and vegetation differences following channel diversion in the Yellow River Delta. Sci. Total Environ. 693, 133600. doi: 10.1016/j.scitotenv.2019.133600 31377360

[B24] Havrdová A. Douda J. Doudová J. (2015). Local topography affects seed bank successional patterns in alluvial meadows. Flora 217, 155–163. doi: 10.1016/j.flora.2015.10.007

[B25] He M. X. Lv L. Y. Li H. Y. Meng W. Q. Zhao N. (2016). Analysis on soil seed bank diversity characteristics and its relation with soil physical and chemical properties after substrate addition. PloS One 11, e0147439. doi: 10.1371/journal.pone.0147439 26808785 PMC4726584

[B26] He M. X. Mo X. Q. Li H. Y. Meng W. Q. (2014). Soil seed bank characteristics and CCA analysis of typical Tianjin coastal saline-alkali wetland. Chin. J. Ecol. 33, 1762–1768. doi: 10.13292/j.1000-4890.2014.0135

[B27] Heerdt G. N. J. T. Verweij G. L. Bekker R. M. Bakker J. P. (1996). An improved method for seed-bank analysis: seedling emergence after removing the soil by sieving. Funct. Ecol. 10, 144–151. doi: 10.2307/2390273

[B28] Hilary F. Angus G. Cai L. Jonathan M. Martin W. S. (2016). Soil stabilization linked to plant diversity and environmental context in coastal wetlands. J. Veg. Sci. 27, 259–268. doi: 10.1111/jvs.12367 27867297 PMC5111397

[B29] Ida T. Y. Harder L. D. Kudo G. (2013). Demand-driven resource investment in annual seed production by a perennial angiosperm precludes resource limitation. Ecology 94, 51–61. doi: 10.1890/12-0619.1 23600240

[B30] Kaiser T. Pirhofer-Walzl K. (2015). Does the soil seed survival of fen-meadow species depend on the groundwater level? Plant Soil. 387, 219–231. doi: 10.1007/s11104-014-2273-8

[B31] Kalamees R. Püssa K. Zobel K. Zobel M.. (2011). Restoration potential of the persistent soil seed bank in successional calcareous (alvar) grasslands in Estonia. Appl. Veg. Sci. 15, 208–218. doi: 10.1111/j.1654-109x.2011.01169.x

[B32] Kalamees R. Zobel M. (2002). The role of the seed bank in gap regeneration in a calcareous grassland community. Ecology 83, 1017–1025. doi: 10.1890/0012-9658(2002)083[1017:trotsb]2.0.co;2

[B33] Kim M. Lee S. H. Lee S. H. Yi K. Kim H. S. Chung S.. (2022). Seed dispersal models for natural regeneration: A review and prospects. Forests 13 (5), 659. doi: 10.3390/f13050659

[B34] Kopáček J. Borovec J. Hejzlar J. Porcal P. (2001). Spectrophotometric determination of iron, aluminum, and phosphorus in soil and sediment extracts after their nitric and perchloric acid digestion. Commun. Soil Sci. Plant Anal. 32, 1431–1443. doi: 10.1081/CSS-100104203

[B35] Li C. J. (2022). Effects of different formulations of soil conditioner on soil fertility enhancement in supplementary cultivated land reconstruction (In Chinese) (Taian: Shandong Agricultural University).

[B36] Li G. Q. Shao W. S. Zhao P. P. Jin C. Q. Chen Y. Y. (2019). Soil seed bank characteristics and soil physicochemical properties of four plant communities in desert steppe region. Acta Ecol. Sin. 39, 6282–6292. doi: 10.5846/stxb201712112227

[B37] Li J. M. Xu H. L. (2009). Distribution pattern of soil seed bank and its relationship with environmental factors in the lower reaches of Tarim River. Soil Water Conserv. 3, 6. doi: 10.13961/j.cnki.stbctb.2009.03.019

[B38] Limón Á. Peco B. (2016). Germination and emergence of annual species and burial depth: Implications for restoration ecology. Acta Oecol. 71, 8–13. doi: 10.1016/j.actao.2016.01.001

[B39] Liu (2005). Research on seed bank in wetland of middle and lower Yangtze River (In Chinese) (Wuhan: Graduate School of Chinese Academy of Sciences (Wuhan Botanical Garden)).

[B40] Ma M. J. Baskin C. C. Yu K. L. Ma Z. Du G. (2017a). Wetland drying indirectly influences plant community and seed bank diversity through soil pH. Ecol. Indic. 80, 186–195. doi: 10.1016/j.ecolind.2017.05.027

[B41] Ma M. J. Dalling J. W. Ma Z. Zhou X. (2017b). Soil environmental factors drive seed density across vegetation types on the Tibetan Plateau. Plant Soil. 419 (1-2), 349–361. doi: 10.1007/s11104-017-3348-0

[B42] Ma M. J. Walck J. I. Ma Z. Wang L. Du G. (2018). Grazing disturbance increases transient but decreases persistent soil seed bank. Ecol. Appl. 28, 1020–1031. doi: 10.1002/eap.1706 29710415

[B43] Ma M. J. Zhou X. H. Du G. Z. (2011). Soil seed bank dynamics in alpine wetland succession on the Tibetan Plateau. Plant Soil. 346, 19–28. doi: 10.1007/s11104-011-0790-2

[B44] Metzner K. Gachet S. Rocarpin P. Saatkamp A. (2017). Seed bank, seed size and dispersal in moisture gradients of temporary pools in Southern France. Basic. Appl. Ecol. 21, 13–22. doi: 10.1016/j.baae.2017.06.003

[B45] Miao S. L. Zou C. B. (2009). Seasonal variation in seed bank composition and its interaction with nutrient enrichment in the Everglades wetlands. Aquat. Bot. 90, 157–164. doi: 10.1016/j.aquabot.2008.08.006

[B46] Middleton B. A. (2016). Effects of salinity and flooding on post-hurricane regeneration potential in coastal wetland vegetation. Am. J. Bot. 103, 1420–1435. doi: 10.2307/44252734 27539261

[B47] Mikajlo I. Pourrut B. Louvel B. Hynšt J. Záhora J. (2023). Plant-soil nitrogen, carbon and phosphorus content after the addition of biochar, bacterial inoculums and nitrogen fertilizer. J. Plant Nutr. 46, 541–555. doi: 10.1080/01904167.2022.2043369

[B48] Miller A. P. Arai Y. (2016). Comparative evaluation of phosphate spectrophotometric methods in soil test phosphorus extracting solutions. Soil Sci. Soc. Am. J. 81, 1543–1550. doi: 10.2136/sssaj2016.08.0256n

[B49] Peterson J. E. Baldwin A. H. (2004). Seedling emergence from seed banks of tidal freshwater wetlands: response to inundation and sedimentation. Aquat. Bot. 78, 243–254. doi: 10.1016/j.aquabot.2003.10.005

[B50] Poschlod P. Rosbakh S. (2018). Mudflat species: Threatened or hidden? An extensive seed bank survey of 108 fish ponds in Southern Germany. Biol. Conserv. 225, 154–163. doi: 10.1111/jbi.12601

[B51] Ren A. T. Hu D. Y. Qi P. X. Zhang S. C. Gao H. M. Mickan B. S.. (2023). Buffering effects of the soil seed bank on annual plant community composition after wetland drying. Land Degrad. Dev. 34, 1601–1611. doi: 10.1002/ldr.4556

[B52] Ruano I. Del Peso C. Bravo F. (2015). Post-dispersal predation of Pinus pinaster Aiton seeds: key factors and effects on belowground seed bank. Eur. J. For. Res. 134, 309–318. doi: 10.1007/s10342-014-0853-z

[B53] Schmiede R. Donath T. Otte A. (2009). Seed bank development after the restoration of alluvial grassland via transfer of seed-containing plant material. Biol. Conserv. 142, 404–413. doi: 10.1016/j.biocon.2008.11.001

[B54] Schneider B. Zilli F. Facelli F. Campana M. (2020). Factors driving seed bank diversity in wetlands of a large river floodplain. Wetlands 40, 2275–2286. doi: 10.1007/s13157-020-01355-9

[B55] Seibert R. Grünhage L. Müller C. Otte A. Donath T. W. (2018). Raised atmospheric CO_2_ levels affect soil seed bank composition of temperate grasslands. J. Veg. Sci. 30, 86–97. doi: 10.1111/jvs.12699

[B56] Stanley E. H. Rojas-Salazar S. Lottig N. R. Schliep E. M. Filstrup C. T. Collins S. M.. (2019). Comparison of total nitrogen data from direct and Kjeldahl-based approaches in integrated data sets. Limnol Oceanogr-Meth. 17, 639–649. doi: 10.1002/lom3.10338

[B57] Thompson K. Fenner M. (2000). The functional ecology of soil seed banks (Wallingford UK: CABI Books).

[B58] Vandvik V. Klanderud K. Meineri E. Måren I. E. Töpper J. (2016). Seed banks are biodiversity reservoirs: species–area relationships above versus below ground. Oikos 125, 218–228. doi: 10.1111/oik.02022

[B59] Wang J. S. Feng J. G. Chen B. X. Shi P. L. Zhang J. L. Fang J. P.. (2016). Controls of seed quantity and quality on seedling recruitment of smith fir along altitudinal gradient in southeastern Tibetan Plateau. J. Mt. Sci-engl. 13, 811–821. doi: 10.1007/s11629-015-3761-x

[B60] Wang S. L. Hu A. Zhang J. Hou F. (2019). Effects of grazing season and stocking rate on seed bank in sheep dung on the semiarid Loess Plateau. Rangeland J. 41, 405–413. doi: 10.1071/RJ19036

[B61] Wang X. H. Li Y. Z. Meng H. Dong H. F. Guo Y. Tong S. Z. (2015). Distribution pattern of new wetland plant communities in Yellow River Delta. Sci. Geogr. Sin. 35, 1021–1026. doi: 10.13249/j.cnki.sgs.2015.08.012

[B62] Wang J. J. Shi B. Yuan Q. Y. Zhao E. J. Bai T. Yang S. P. (2022). Hydro-geomorphological regime of the lower Yellow River and delta in response to the water–sediment regulation scheme: Process, mechanism and implication. Catena 219, 106646. doi: 10.1016/j.catena.2022.106646

[B63] Wei M. Q. Zhao L. J. Qiu G. L. Su Z. N. Tan S. Y. (2017). Spatial distribution and biological influencing factors of soil seed banks of *Halophilus bakerii* in intertidal zone (*In Chinese*). J. GuangXi Acad. Sci. 33, 93–101. doi: 10.13657/j.cnki.gxkxyxb.20170428.001

[B64] Yu J. B. Zhan C. Li Y. Z. Zhou D. Fu Y. Q. Chu X. J.. (2016). Distribution of carbon, nitrogen and phosphorus in coastal wetland soil related land use in the Modern Yellow River Delta. Sci. Rep. 6, 37940. doi: 10.1038/srep37940 27892492 PMC5124950

[B65] Zhang M. Chen F. Q. Chen S. H. Xie Z. Q. Huang Y. W. Liu W. B.. (2017). The soil seed bank of a rehabilitated draw-down zone and its similarity to standing vegetation in the Three Gorges Reservoir Area. Ecol. Res. 32, 1011–1021. doi: 10.1007/s11284-017-1518-4

[B66] Zhao Y. P. Liao J. C. Bao X. K. Ma M. J. (2021). Soil seed bank dynamics are regulated by bird diversity and soil moisture during alpine wetland degradation. Biol. Conserv. 263, 109360. doi: 10.1016/j.biocon.2021.109360

[B67] Zhao X. X. Tian Q. X. Michelsen A. Lu M. Z. Ren B. S. Huang L.. (2023). The effect of experimental warming on fine root functional traits of woody plants: Data synthesis. Sci. Total Environ. 894, 165003. doi: 10.1016/j.scitotenv.2023.165003 37348713

[B68] Zhao Y. T. Wang G. D. Zhao M. L. Wang M. Jiang M. (2022). Direct and indirect effects of soil salinization on soil seed banks in salinizing wetlands in the Songnen Plain, China. Sci. Total Environ. 819, 152035. doi: 10.1016/j.scitotenv.2021.152035 34856265

[B69] Zhu Z. C. Slangen A. Zhu Q. Gerkema T. Bouma T. J. Yang Z. F. (2022). The role of tides and winds in shaping seed dispersal in coastal wetlands. Limnol. Oceanogr. 67, 646–659. doi: 10.1002/lno.12024

[B70] Zou C. Martini F. Xia S. W. Castillo-Diaz D. Goodale U. M. (2021). Elevation and micro environmental conditions directly and indirectly influence forests’ soil seed bank communities. Glob. Ecol. Conserv. 26, e01443. doi: 10.1016/j.gecco.2020.e01443

